# Developing a validity testing plan for a patient-reported outcome measure in a new context using the argument-based approach to validity

**DOI:** 10.1007/s11136-026-04269-x

**Published:** 2026-06-06

**Authors:** Scott W. Gill, Christina Cheng, Julia Bowman, Gerald Elsworth, Caron Shaw, Richard H. Osborne, Melanie Hawkins

**Affiliations:** 1 Global Health and Equity Development Hub, La Trobe University, Melbourne, Australia; 2 Research Unit, Justice Health and Forensic Mental Health Network, Sydney, Australia; 3 Corporate Services, Clinical Excellence Commission, Sydney, Australia; 4 Department of Public Health, University of Copenhagen, Kobenhavn, Denmark

**Keywords:** Validity testing plan, Patient-reported outcome measures, Argument-based approach to validity, Health Literacy Questionnaire, HLQ, Validity, Prisons

## Abstract

Health-related measures, such as Patient-Reported Outcome Measures (PROMs), are widely used in clinical practice, research, service evaluation, and decision-making. Validity is about the extent to which a score interpretation is supported by evidence for a specific use in a specific context. The argument-based approach to validity provides a systematic way of planning and undertaking validity testing and is gaining attention in health-related measurement, yet few practical examples of its application exist. This commentary firstly describes the phases and steps of the argument-based approach to validity. Secondly, it brings the theory into practice by presenting a worked example that illustrates how to apply the argument-based approach to validity to develop a validity testing plan for the application of a widely used health literacy PROM in a new context. Importantly, the validity testing plan outlines the underpinning assumptions that need investigation to determine the extent to which we can have confidence in the interpretation and use of Health Literacy Questionnaire (HLQ) scores in the New South Wales prison context. The commentary offers a practical exemplar for identifying assumptions, evidence needs and methodological options to support validity testing and to develop an evidence-based argument to verify score interpretation and use in a specific context. The validity testing planning process described and demonstrated in this paper will support the uptake of this approach and promote scientific rigour in health-related measurement.

**Supplementary Information:**

The online version contains supplementary material available at 10.1007/s11136-026-04269-x.

## Introduction

Health-related measures, such as Patient-Reported Outcome Measures (PROMs), are widely used in clinical practice, research, service evaluation, and decision-making [1–4]. To date, validation practices in health tend to lead to statements about health-related measures being “valid” [5]. However, evidence from one context may not justify or be relevant for a measure’s use for other purposes or in other settings, and score interpretation may potentially lead to inappropriate decision making in the new context [5, 6]. The settings and cultures in which people live are likely to impact how people interpret and respond to questionnaire items; therefore, it is important not to assume that people understand, engage with and respond to items in the same way in different contexts [7]. Equitable, unbiased measurement relies on the extent to which we have confidence that score interpretation in a new context has the evidence to support it for the intended decision making use [5, 8, 9]. The argument-based approach to validity [90, 99, 100, 23] provides a systematic way to explicitly state the intended interpretation and use of a measure's scores and to outline the assumptions that underpin them. This approach, in turn, supports targeted validity testing by identifying the relevant sources of validity evidence that need to be generated and evaluated, thus reducing research waste.

In a companion paper to this paper, Hawkins and colleagues [5] describe the theoretical foundations of the argument-based approach to validity in detail. Complementing the Hawkins et al. paper, this paper has two aims. First, it reiteratires the phases and steps of the argument-based approach to validity. Second, it brings the theory into practice by presenting a worked example of how to apply the argument-based approach to develop a validity testing plan to investigate the use of a health literacy measure in a prison context.

## Planning the validation process

Validation practices and validity theory have evolved over the past century [10, 11], with the 2014 Standards for Educational and Psychological Testing (hereafter, the *Standards*) [12] recognised as the authoritative reference in education and psychology [11]. The *Standards* define validity as “the degree to which evidence and theory support the interpretations of test scores for proposed uses of tests” [12, p. 11] and describe validation as a process that “involves accumulating relevant evidence to provide a sound scientific basis for the proposed score interpretations” [12, p. 11]. Thus, validity is a continuum, not a binary label, that reflects the extent to which evidence provides confidence in the interpretation and use of a measure’s scores [8, 9, 13]. A validation process requires the planning for and gathering of relevant evidence about the interpretation and use of a measure’s scores in a particular context [8, 9, 13, 14].

The *Standards* indicate that validity testing is a process of constructing and evaluating arguments for and against the intended interpretation and use of a measure within a context [12]. The argument-based approach to validity is both a methodological and practical way to undertake validity testing [11] and has been gaining attention in health-related measurement [10, 14–21]. The initial steps involve clearly stating the proposed interpretation and use of scores derived from a measure and articulating a set of propositions (i.e., assumptions) that underpin the interpretation and use [12]. The assumptions then inform decisions about the types of evidence needed and appropriate methods to generate that evidence [12]. Collectively, these steps shape the groundwork for a systematic validation plan, ensuring evidence generation and collection not only align with but are logically organised to justify the intended interpretation and use of a measure's score in context.

## The argument-based approach to validity

**Fig. 1:**
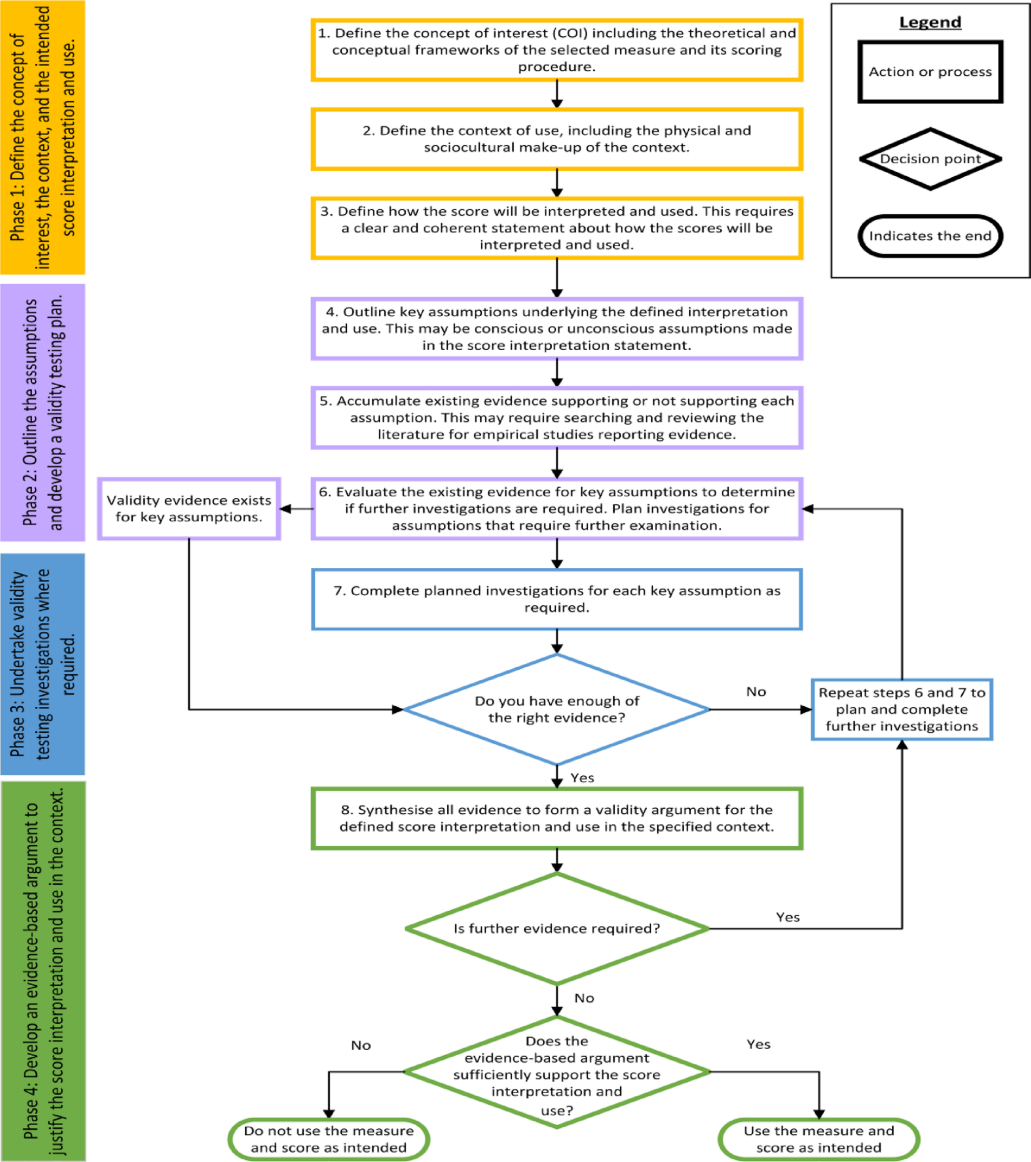
The phases and steps involved in the argument-based approach to validity. *Note*: Each decision point and its associated pathways require professional judgement based on the specific measure, the context, and the intended interpretation and use. In some cases, a measure may be used with caveats if professionally justified.

In the context of health-related measures, the approach can be broken down into four distinct phases and eight steps (Fig. 1). The approach is not a linear process and requires professional judgement based on the methods employed in the field relevant to the measure’s application [12].

### Phase 1: Define the concept of interest, the context, and the intended score interpretation and use

#### 1. Define the Concept of Interest and its measurement

The concept (or construct) of interest (CoI) (i.e., what is being measured) needs to be clearly understood and defined. A concept’s definition largely determines how it is measured. It is, therefore, essential to understand the theoretical and conceptual frameworks of the selected measure and its scoring procedure.

#### 2. Define the Context of Use

The context of use—that is, the physical and sociocultural make-up of the context [22]—needs to be clearly defined and understood. This may include, but is not limited to, the population of interest and sub-groups that may exist within the population, external environmental factors, organisational dynamics and structures, resources, leadership, political intersections, and societal views [22].

#### 3. Define how the score will be interpreted and used in the context

Make a clear and coherent statement about how the scores derived from a measure will be interpreted and used within the context. This statement forms the basis of the validation process, enabling underpinning assumptions to be outlined and a validity testing plan to be detailed.

### Phase 2: Outline the assumptions and develop a validity testing plan

#### 4. Outline key assumptions underlying this interpretation and use

Based on the defined score interpretation and context, key assumptions underpinning the intended use need to be outlined to avoid misinterpretation of scores and potentially harmful, unfair, or inequitable health consequences. Many assumptions are made unconsciously when using a measure, whilst some may be more explicit, depending on the intended interpretation and use [5]. Some common assumptions for health-related measures have been detailed elsewhere [5, 16]. However, these should not be used as a checklist, as other assumptions may need to be considered.

#### 5. Accumulate evidence supporting or not supporting each assumption

Validity evidence needs to be accumulated for each assumption. Search and review the literature for empirical studies that have generated and reported validity evidence relevant to the CoI, score interpretation, and the context of use. This evidence may support or not support the assumptions (i.e., contradict or nullify the result).

#### 6. Evaluate which, if any, key assumptions require further investigation and then plan investigations into these assumptions

Evaluate if the accumulated validity evidence adequately supports each key assumption underlying the measure’s intended interpretation and use. If no or limited validity evidence exists or if unclear or uncertain evidence (i.e., findings from studies contradict each other) has been accumulated, identify appropriate methods for further testing. Then plan investigations to generate evidence about the assumption.

### Phase 3: Undertake validity testing investigations where required

#### 7. Complete planned investigations

Undertake the planned validity testing for each key assumption as required. As new evidence is generated, professional judgment is necessary to determine whether additional evidence is needed [12]. Steps 6 and 7 can be repeated as necessary to continue generating and accumulating evidence 

### Phase 4: Develop an evidence-based argument to justify the score interpretation and use in the context

#### 8. Develop the evidence-based argument for the score interpretation and use in the context

Synthesise all accumulated validity evidence, in relation to the assumptions and construct theory, to establish the extent to which these sources of evidence support or do not support the intended score interpretation and use in the specified context [11, 23]. The evidence-based argument requires judgement to consider, based on the evidence and professional experience, the extent to which the score interpretation is appropriate, meaningful, and useful for the intended use in the specified context. That is, the extent to which a confident argument can be made about the validity of the score interpretation and use in context.

## Sources of validity evidence

The *Standards* [12] describe five sources of validity evidence: evidence based on 1) measure content, 2) response processes, 3) internal structure, 4) relations to other variables, and 5) validity-related consequences of measurement (Table 1). The five sources of evidence provide a framework to plan, gather and report appropriate sources of evidence from validity testing studies [20]. The intended interpretation and use statement, and the assumptions inherent in it, guide developers as to which sources of validity evidence are appropriate and relevant to their use of a measure in a specific context. Combining both the sources of evidence and assumptions underpinning the intended interpretation and use will guide researchers in highlighting the relevant methods for validity testing. 

**Table 1:** The five sources of validity evidence

The five sources of validity evidence
1. Evidence based on measure content
The relationship of the item themes, wording, and format with the intended construct, including administration (e.g., data collection methods such as mail-out, face-to-face interview or online survey) and scoring procedures (e.g., response options and scoring methods).
2. Evidence based on response processes
The cognitive processes and interpretation of items by respondents and users, as measured against the intended construct. Including “what people do, think, or feel when interacting with, and responding to, the item” [24, p. 2].
3. Evidence based on internal structure
The extent to which item interrelationships conform to the intended construct. For example, the Health Literacy Questionnaire’s nine-scale structure and items conforming to each of the nine scales intended by the developers.
4. Evidence based on relations to other variables
The pattern of relationships of a measure's scores to theoretically relevant external variables as predicted by the intended construct.
5. Evidence for validity-related consequences of measurement
Intended and unintended consequences, whether negative or positive, can be related to validity. Negative consequences can signal a source of invalidity, such as construct under-representation or construct-irrelevant variance.

*Source:* Modified from Hawkins, Elsworth [11], American Educational Research Association [12], Hawkins, Elsworth [18].

## An example of developing a validity testing plan for the Health Literacy Questionnaire (HLQ) in the New South Wales (NSW) prison context

Below, this commentary presents a worked example to demonstrate how the argument-based approach using the *Standard's* validity evidence framework can be used to develop a validity testing plan. The example is the Health Literacy Questionnaire (HLQ) being used in the New South Wales (NSW), Australia, prison context. As this is only a plan, Phases 3 and 4 fall outside the scope of this commentary.

### Phase 1: Define the concept of interest, the context, and the intended score interpretation and use

#### Health literacy and the Health Literacy Questionnaire (HLQ)

Health literacy is a complex, multi-dimensional concept [25] that represents how individuals or communities access, understand, appraise, remember, and use health information and services [26]. The HLQ [25] is a widely used health literacy measure, that has been applied in over 80 countries to understand people’s health literacy strengths and challenges.

The HLQ was initially developed and tested in an Australian health context using a grounded approach [25]. It comprises nine independent scales that were generated through co-design processes with community members and practitioners [25]. Each scale has from 4 to 6 items (total of 44 items). The first five scales employ a four-point response option scale from (1 = *Strongly disagree* to 4 = *Strongly agree)* and the remaining four scales employ a five-point response option scale (1 = *Cannot do or always difficult* to 5 = *Always easy)*. Scores for each HLQ scale are calculated by summing all items in a scale and dividing by the number of items, resulting in nine scale scores (explicit equal weighting) [25]. Table 2 displays the intended meanings of high and low scoring for each scale construct [27].

**Table 2:** Descriptions of the intended meanings of the nine HLQ scale constructs represented by high and low scores for each construct

Lower scores on the construct	Higher scores on the construct
Part 1: Scales 1 to 5 Score range: 1 to 4 (1 = Strongly disagree, 2 = Disagree, 3 = Agree, 4 = Strongly agree)	
1. Feeling understood and supported by healthcare providers (4 items)	
Is unable to engage with doctors and other healthcare providers. The person doesn’t have a regular healthcare provider and/or has difficulty trusting healthcare providers as a source of information and/or advice.	Has an established relationship with at least one healthcare provider who knows them well and who they trust to provide useful advice and information and to assist them to understand information and make decisions about their health.
2. Having sufficient information to manage my health (4 items)	
Feels that there are many gaps in their knowledge and that they don’t have the information they need to live with and manage their health concerns.	Feels confident that they have all the information that they need to live with and manage their condition and to make decisions.
3. Actively managing my health (5 items)	
Doesn’t see their health as their responsibility, they are not engaged in their healthcare and regard healthcare as something that is done to them.	Recognise the importance of and are able to take responsibility for their own health. They proactively engage in their own care and make their own decisions about their health.
4. Social support for health (5 items)	
Completely alone and unsupported.	A person’s social system provides them with all the support they want or need.
5. Appraisal of health information (5 items)	
No matter how hard they try, they cannot understand most health information and get confused when there is conflicting information.	Is able to identify good information and reliable sources of information. They can resolve conflicting information by themselves or with help from others.
Part 2: Scales 6 to 9 Score range: 1 to 5 (1 = Cannot do or always difficult, 2 = Usually difficult, 3 = Sometimes difficult, 4 = Usually easy, 5 = Always easy)	
6. Ability to actively engage with healthcare providers (5 items)	
Is passive in their approach to health care, inactive, i.e., they do not proactively seek or clarify information and advice and/or service options. They accept information without question. Unable to ask questions to get information or to clarify what they don’t understand. They accept what is offered without seeking to ensure that it meets their needs. Feel unable to share concerns.	Is proactive about their health and feels in control in relationships with healthcare providers. Is able to seek advice from additional health care providers when necessary. They keep going until they get what they want. Empowered.
7. Navigating the healthcare system (6 items)	
Is unable to advocate on their own behalf and unable to find someone who can help them use the healthcare system to address their health needs. Do not look beyond obvious resources and have a limited understanding of what is available and what they are entitled to.	Is able to find out about services and supports so they get all their needs met. Able to advocate on their own behalf at the system and service level.
8. Ability to find good health information (5 items)	
Cannot access health information when required. Is dependent on others to offer information.	Is an “information explorer”. Actively uses a diverse range of sources to find information and is up to date.
9. Understanding health information well enough to know what to do (5 items)	
Has problems understanding any written or spoken health information or instructions about treatments or medications. Unable to read or write well enough to complete medical forms.	Can understand all written and spoken information (including numerical information) in relation to their health and able to write appropriately on forms where required.

*Source*: Adapted with permission from Osborne et al. [27].

A wide range of validity evidence has accumulated globally about the interpretation and use of HLQ scores, demonstrating it to be a robust measure for use in a broad range of contexts such as disease-specific research [28–36], community-based health research [25, 37–46], research with older people [47, 48], caregivers [49], and university students [50–52]. Despite this, no evidence exists to support the interpretation and use of HLQ data in the prison context [53]. For example, someone in prison may experience institutionalisation [54, 55] given that prisons are designed to restrict an individual's liberty [56], resulting in individuals having little to no control over their daily routine or freedom to make choices. This power imbalance may lead people in prison to respond in ways they believe will appease authorities rather than reflect their personal views. Therefore, validity evidence is required to understand the extent to which inferences drawn from HLQ scores are valid for decision-making purposes in this context.

#### The New South Wales (NSW) prison context

Prisons are institutions of the criminal justice system and are inherently restrictive contexts [56]. The state of NSW in Australia has 34 adult prisons, with 32 publicly run by Corrective Services NSW (CSNSW) [57]. CSNSW maintains the security and order of the prison system, whereas the Justice Health and Forensic Mental Health Network (Justice Health NSW), a statutory health corporation [58], delivers healthcare to people in prison. As delineated in the United Nations Nelson Mandela Rules [59], the NSW public health system is required to provide services equivalent to those available in the community. To meet these requirements, Justice Health NSW offers a range of nursing, medical and allied health services, with service provision varying across prisons [60]. People in prison access health services through self-referral, routine or reception screening and follow-up appointments, with acute care provided in local public community health services.

People in prison are often from populations experiencing marginalisation and tend to have complex healthcare needs. NSW has Australia’s largest prison population, with over 13,000 people in prison [61]. The prison population is mainly male (92%), is relatively young (median age 36 years), and the representation of Aboriginal and Torres Strait Islander people is 10 times higher than the general population [61–63]. People in NSW prisons also experience higher rates of communicable and non-communicable diseases compared with the general Australian population [64–68]. Given their complex health needs, understanding and responding to the health literacy strengths and challenges of the prison population could potentially improve their health outcomes.

#### The intended Health Literacy Questionnaire (HLQ) scale score interpretation and use in the New South Wales (NSW) prison context

HLQ scores are interpreted according to the HLQ item intents [69] (also known as concept definitions [70]) and high/low scale construct descriptors (see Table 2 above). The nine scale scores reveal a profile of health literacy strengths and challenges, which are commonly used in surveys, evaluation and as a needs assessment in the Ophelia (Optimising Health Literacy and Access) process [71, 72] to inform the development of interventions.

Based on this intended use, HLQ score interpretation is defined for the current example in Box 1.

Box 1: The intended interpretation and use of the nine Health Literacy Questionnaire (HLQ) scale scores in the New South Wales (NSW) prison context

**Table:** 

HLQ scale scores will provide data about the health literacy strengths and challenges of adults in prison. These scores will be interpreted according to the item intents and high/low descriptors of the HLQ scales, as described by the HLQ authors [25]. Correctly calculated scale scores will indicate the health literacy strengths and challenges of different characteristic groups of people in prisons. Healthcare decision-makers can use these scale scores to:	
a.	Describe the health literacy profiles of people in prison, and
b.	Support decision making about action ideas for intervention development that will improve access to health services and information and remove healthcare barriers for people in prison.

#### Phase 2: Outline the assumptions and develop a validity testing plan

Based on the CoI and the HLQ score interpretation and use in the NSW prison context, six assumptions were identified (Table 3). Table 3 outlines a validity testing plan for the HLQ score interpretation and use in the NSW prison context. The evidence needed and methods that could be used for evidence generation and evaluation are described below in relation to each assumption.

**Table 3:** A validity testing plan for the health literacy questionnaire (HLQ) score interpretation and use in the New South Wales (NSW) prison context

Assumptions	What the evidence tells us	Methods to generate evidence	Type of evidence
1. People in prison understand and respond to the HLQ items as intended by the developers (i.e., as per the authors’ descriptions of HLQ item intents), and the items reflect their lived experiences.	1.1 The item response options for HLQ Part 1 (4-point agreement scale) and Part 2 (5-point difficulty scale) are appropriate for people in prison. 1.2 People in prison understand, engage with, and respond to the items as intended. 1.3 People in prison select item responses that meaningfully reflect their daily experiences and the intent of the items.	Cognitive interviews with people in prison – using the methods employed by Leslie et al. [49] to elicit narratives from respondents about their responses to items. Thematic analysis of cognitive interview narratives to determine the extent of match and no match between respondent narrative data about reasons for response selections (i.e., item scores). It will also assess concordance and discordance between respondent narrative data and the item intent descriptors provided by the HLQ authors [49].	Measure content Response processes
2. The HLQ nine-scale conceptual structure of health literacy is replicated in the prison context.	2.1 The HLQ data from people in NSW prisons replicate and fit the nine one-factor models and the nine-scale conceptual structure of health literacy.	Confirmatory Factor Analysis using Bayesian Structural Equation Modelling [73].	Internal structure
	2.2 There is conceptual discrimination between the nine HLQ scales, as hypothesised a priori, indicating each scale is theoretically distinct from the other eight scales.	Discriminant validity, using the Fornell-Larcker criteria [74].	
3. Items within each of the nine HLQ scales are interrelated, such that they provide internally consistent scale scores (reliability).	3.1 HLQ scales demonstrate internal consistency reliability.	Composite Scale Reliability calculated using Raykov’s method [75] and Cronbach’s alpha [76].	Reliability^a^
4. HLQ items reflect a range of health literacy capabilities and are sensitive to identify people with different abilities for each scale construct.	4.1 HLQ items exhibit an ordered set of response thresholds, item discrimination levels, and measure a range of the scale's construct.	Item Response Theory by applying a generalised partial credit model (GPCM) to the data and examining the item characteristic curves and information curve [77].	Internal structure
	4.2 Items within each scale demonstrate a range of difficulty, with a variation in the proportion of respondents negatively endorsing an item as against positively endorsing an item.	Calculate item difficulty as the proportion of respondents negatively (i.e., Strong disagree or Cannot do or always difficult), endorsing an item, as against positively (i.e., select Strongly agree or Always easy) [78].	
5. HLQ scale scores demonstrate measurement equivalence across subgroups and settings within the prison context.	5.1 Measurement equivalence is demonstrated for scores across the nine HLQ scales across subgroups (i.e., sex, Aboriginal identity, legal status, age, and education level) and settings (i.e., security classification) within the prison context.	Measurement invariance analysis using the Bayesian alignment methods [79].	Internal structure
6. Scores from the nine HLQ scales can be used as a needs assessment to inform the development of interventions in the NSW prison context.	6.1 The nine HLQ scale scores can be used to develop a range of health literacy profiles of people in NSW prisons.	Cluster analysis of HLQ data combined with demographic and health data and data from semi-structured interviews with people in prison to inform the development of vignettes, as described in the Ophelia Manual [27].	Validity-related consequences of measurement
	6.2 The vignettes appropriately portray the lived experiences of different groups of people in the prison population, according to the nine HLQ scale high/low descriptors.	Vignette checking by key stakeholders (e.g., people in prison, healthcare and prison workers) verifies that the personas depicted resonate with their experiences of the strengths, challenges and characteristics of people accessing the health services in NSW prisons. In ideas generation workshops and yarning circles participants are asked a guiding question like “Do you see people like this person in your centre?” [80], to verify each vignette's persona.	
	6.3 Vignettes generated from the HLQ scale scores can be used to generate action ideas for intervention development.	Thematic analysis to identify the action ideas generated and rating of individual ideas by staff on their importance, current implementation and feasibility in the NSW prison context.	

^a^The *Standards* does not recognise reliability as a source of validity evidence. The *Standards* state “that the level of reliability/precision of scores has implications of validity” [12, p. 34] and that “appropriate evidence of reliability/precision should be provided for the interpretation for each intended score use” [12, p. 42]. Therefore, reliability has been included to provide further evidence for the HLQ’s conceptual framework.

##### Assumption 1: People in prison understand and respond to the HLQ items as intended by the developers, and the items reflect their lived experiences.

Evidence is needed to evaluate three key components of this assumption. First, whether the HLQ item response options are appropriate. Second, whether people in prison understand, engage with and respond to the items as intended (i.e., as per the authors’ descriptions of HLQ item intents [69]). Third, whether their HLQ responses reflect their lived experiences and the intended meaning of each item. Cognitive interviews can be used to generate *response processes* and *measure content* evidence. This process typically involves an interviewer reading each HLQ item aloud. Respondents then mark their response on their version of the questionnaire. Following this, the interviewer may ask respondents:

1. "Why did you choose this answer?"
2. "What thoughts came to mind that helped you answer this question?"

This process can be repeated for all HLQ items.

Interviews can then be transcribed verbatim, and narrative data can be analysed using a deductive thematic approach [81], as described by Leslie and colleagues [49]. This usually involves three steps:

1. Comparing participant item responses with their narrative data about why they selected that answer to determine if the item response and narrative match.
2. Comparing participant narrative data about each of their item responses with the HLQ item intent descriptions to determine if there is concordance.
3. Conducting a thematic analysis to ascertain the reasons why item responses don't match narrative data (Step 1) or there is discordance between participant narrative data and HLQ item intent descriptions (Step 2).

Step 1 provides *measure content and response processes* evidence for the HLQ item response options. Step 2 generates evidence for whether items are understood and engaged with as intended. The thematic analysis helps determine the concordance and discordance, enabling the generation and evaluation of the *measure content and response processes* evidence for Assumption 1.

##### Assumption 2: The HLQ nine-scale conceptual structure of health literacy is replicated in the prison context.

*Internal structure* evidence is required to demonstrate if HLQ data from people in prison replicate the nine one-factor models and the HLQ nine-scale conceptual structure. Confirmatory Factor Analysis (CFA) can be used to test whether people’s responses to HLQ items within each scale align with the *a priori* nine one-factor models and HLQ’s nine-scale model (Supplementary Fig. 1) [82]. A Bayesian Structural Equation Modelling (BSEM) approach to CFA offers greater flexibility by using informative priors to allow small cross-loadings and residual correlations, which can help the model better reflect the theory it is based on [73, 78]. The BSEM approach to CFA can generate evidence on whether the *a priori* nine one-factor and nine-factor structure of the HLQ is appropriate for responses in the prison context or whether simpler (or more complex) models would better fit the data [83]. To investigate unidimensionality, a series of one-factor models can be applied to each of the nine scales of the HLQ, before testing for the nine-factor model. The BSEM results can provide *internal structure* evidence to evaluate Assumption 2.

*Internal structure* evidence is also required to evaluate if the nine HLQ scales are discriminant from each other, as per the *a priori* nine-scale HLQ structure. The Fornell-Larcker criteria [74, 84] can be used to examine discriminant validity, which refers to the extent to which a construct is distinct from other constructs [85]. Discriminant validity is supported when relationships between distinct latent variables (i.e., the nine HLQ scales) are small to moderate [83]. Using a structural equation modelling program such as Mplus [82], the Fornell-Larcker criteria can provide evidence of whether the nine HLQ scales are discriminant from one another. The results can generate *internal structure* evidence for discriminant validity to evaluate Assumption 2.

##### Assumption 3: Items within each of the nine HLQ scales are interrelated, such that they provide internally consistent scale scores (reliability).

Evidence is required to determine if HLQ scales demonstrate internal consistency reliability. Internal consistency reliability is a property of a scale that reflects the inter-item covariance among individual items contributing to a summated scale score [85]. Composite scale reliability can be calculated using statistical software such as Mplus [82] by applying Raykov’s method [75], noting criticism of Cronbach’s coefficient alpha in producing potentially biased estimates [25, 76, 78]. Cronbach’s alpha, calculated using SPSS [86], may be included for comparison with earlier HLQ studies. Both estimates are comparable when the construct is unidimensional, has uncorrelated errors, and has uniformly high loadings on the underlying true score [75, 87]. Both reliability estimates can provide evidence to determine if HLQ scales demonstrate internal consistency reliability, such that scale scores are reliable.

##### Assumption 4: HLQ items reflect a range of health literacy capabilities and are sensitive to identify people with different abilities for each scale construct.

Evidence is needed to determine if the HLQ items exhibit an ordered set of response thresholds and item discrimination levels (i.e., how well an item differentiates between individuals with different trait levels [77]), and if the items measure a range of the scale’s construct. Item Response Theory (IRT) is a type of measurement theory that explores the relationship between how an individual responds to items on a scale and their abilities [77, 88]. If the IRT assumptions are met, a Generalized Partial Credit Model (GPCM) can be applied using software such as IRTPRO [89]. A GPCM is well suited due to the HLQ’s Likert-type scales and the flexibility of the GPCM, which allows for the estimation of both discrimination and category response parameters for each item [77]. For each HLQ item, the item characteristic curve can be examined. Item thresholds can be examined to evaluate if each HLQ item exhibits an ordered set of response thresholds for each response category. The slope of categorical response curves (CRC) can be examined to evaluate item discrimination levels [77]. The item location and information curve can be examined to evaluate if HLQ items measure a range of each scale’s construct. The results of the IRT analysis using a GPCM would support the evaluation of *internal structure* evidence for Assumption 4.

Evidence is required to examine if the 44 HLQ items have differing levels of difficulty as intended by the authors [25]. Item difficulty can be examined by combining HLQ item response options to form dichotomous indicators [25]. The four HLQ Part 1 response options (*Strongly disagree*, *Disagree*, *Agree*, *Strongly agree*) will be dichotomised to Disagreement and Agreement. The five HLQ Part 2 response options will be dichotomised to Difficult (*Cannot do or always difficult*, *Usually difficult*, *Sometimes difficult*) and Easy (*Usually easy*, *Always easy*). The ratio of participants who endorse items positively (Agreement or Easy) and negatively (Disagreement or Difficult) can be calculated. Item difficulty calculations provide *internal structure* evidence to evaluate Assumption 4. Moreover, the dichotomous difficulty calculations can complement the IRT item location parameters, which could support a more robust assessment of Assumption 4.

##### Assumption 5: HLQ scale scores demonstrate measurement equivalence across subgroups and settings within the prison context.

Evidence is required to determine measurement invariance for HLQ items and scales across population sub-groups and settings to ensure that items are interpreted and responded to in the same way across these groups and settings. Measurement invariance approaches assess the equivalence of a construct (e.g., health literacy) across defined groups [91]. Measurement invariance can be investigated in software such as Mplus using the alignment method described by Asparouhov and Muthen [79], which offers an alternative to the traditional partial invariance approach for estimating comparable factor means when full invariance is violated [92]. The alignment method compares latent factor means and variances across groups without requiring exact measurement invariance (i.e., full invariance) [39, 79, 93, 94]. The fit of the alignment model to the data is equivalent to that of the chosen configural model [93].

Using a Bayesian estimation approach, the alignment approach can be employed to examine measurement equivalence across subgroups (i.e., sex, Aboriginal identity, legal status, age, and education level), and settings (i.e., security classification). These subgroupings have been selected because they may influence individuals’ responses to HLQ items. For instance, an individual’s legal status (i.e., remanded or sentenced) may affect the types of services they have access to in prison [53]. Similarly, their security classification (i.e., minimum, medium or maximum) may determine their level of access to services [95], which could impact how they respond to specific items. These results would generate *internal structure* evidence to evaluate Assumption 5.

##### Assumption 6: Scores from the nine HLQ scales can be used as a needs assessment to inform the development of interventions in the NSW prison context.

Evidence is needed to evaluate if the HLQ data generated from the prison population can be used as a needs assessment to inform the development of interventions. Three key components of evidence are required to evaluate Assumption 6. First, whether the nine HLQ scale scores can be used to develop a range of health literacy profiles of people in NSW prisons. Second, whether the vignettes (i.e., evidence-based stories) developed from HLQ profiles appropriately portray the lived experiences of groups of people in the prison population, according to the nine HLQ scale high/low descriptors. Third, whether vignettes generated from HLQ scale scores from people in prison can be used to generate needed, wanted, and feasible action ideas for intervention development. The Ophelia process [71, 72] is a widely used methodology that is used to co-design health literacy-informed interventions, with its utility being demonstrated in a range of countries and settings [96, 97]. The methods used within the process may generate this evidence.

The Ophelia process employs hierarchical cluster analysis using Ward’s method with HLQ, demographic and health data from people in prison. The Ophelia cluster analysis can be undertaken to identify groups of respondents with similar patterns of HLQ scores across the nine separately scored scales. Semi-structured interviews can be conducted with people in prison to gather contextual data about their prison healthcare experiences and explore why they selected scores on HLQ scales [80]. The cluster analysis results and data from the semi-structured interviews with people in prison can be used to inform the development of vignettes.

Developed vignettes can then undergo checking by key stakeholders (e.g., people in prison, healthcare, and prison workers) to verify that the personas depicted align with their experiences of the strengths, challenges and characteristics of people accessing the health services in NSW prisons. Further vignette checking can occur in ideas generation workshops and yarning circles, where participants will be asked a guiding question, like “Do you see people like this person in your centre?”, or “Do you think there are people like this around you in prison?” [80], to verify the personas in each vignette before being asked to suggest actions and solutions to address identified health literacy challenges.

Thematic analysis of the actions and solutions suggested by ideas generation workshop and yarning circle participants can identify the action ideas generated. Health and corrections staff may then be invited to rate each action idea for importance, current implementation status, and what might be feasible for implementation. Applying methods used in the Ophelia process to HLQ data from the NSW prison context may provide *validity-related consequences of measurement* evidence to evaluate Assumption 6.

## Preparing for Phases 3 and 4: Operationalising the validity testing plan

The validity testing plan outlines a methodical approach to generating evidence for each assumption, enabling the application of Phases 3 and 4 of the argument-based approach (not described in this commentary). This ensures that assumptions are critically evaluated before HLQ data are used to support decision-making about interventions designed to improve access to health services and information and to remove healthcare barriers for people in prison. This is particularly important given the well-documented vulnerabilities and poorer health outcomes experienced by people in prison [64–68] and to minimise potential epistemic injustices [7, 53]. Although health literacy development has the potential to support service reform and improve health outcomes of this population [26, 53], there is currently a lack of validity evidence supporting this intended use of the HLQ. Operationalising this validity testing plan has the potential to generate the first validity evidence for the HLQ in this context and also contribute the first evidence for its use to support health services improvement in prisons, which is highly relevant to prison health authorities globally.

The example described in this paper identified six key assumptions that underpin the intended interpretation and use of HLQ scores in the NSW prison context. These assumptions span from the item-level to broader conceptual structure and include the application of the HLQ as a needs assessment to inform intervention development. The development of this example validity testing plan identified the need to undertake mixed-methods investigations to build a logical evidence-based argument for the HLQ’s intended interpretation and use in the NSW prison context. The validity testing plan outlines methods that can be used to generate and evaluate evidence for each assumption. Due to the iterative nature of the argument-based approach to validity, as new validity evidence is generated and evaluated, professional judgement would be required to determine whether alternative or additional methods are needed [12]. This judgement can ensure the critical evaluation of each assumption for the intended interpretation and use [12]. In turn, it can enable the synthesis of all accumulated evidence, providing a strong scientific basis for developing a logical evidence-based argument about the extent to which HLQ score interpretations are valid to inform intervention development in the NSW prison context.

## Implications

This paper provides a clear example of how to develop a validity testing plan. The description of the argument-based approach to validity and the accompanying flowchart (Fig. 1) outline a step-by-step process to support the uptake and operationalisation of this approach in practice. The argument-based approach is increasingly being applied in health-related measurement [10, 14–21] and as highlighted by Weinfurt, it “is strongly reflected in US Food and Drug Administration’s (FDA) Patient Patient-Focused Drug Development draft Guidance 3” [10]. This new FDA guidance [98] represents a regulatory shift, encouraging measure users to explicitly state their intended interpretation and use of clinical outcome assessments, such as patient-reported outcomes (including PROMs and quality of life measures) [98] and the assumptions underpinning these statements. Furthermore, the guidance calls for measure users to plan relevant investigations to generate targeted, context-relevant validity evidence to support their claims. The argument-based approach addresses current challenges faced about the amount and type of evidence required to justify the use of a measure in a new context [10]. The uptake of the argument-based approach in health-related measurement will move validation practices away from checklist-driven or quantity-over-quality approaches to gathering evidence to answer specific validity questions [16]. In other words, developing a validity testing plan brings a new level of rigour to health-related measurement, where explicit assumptions, methods, and protocols related to the intended score interpretation and use in context are outlined before validity testing is undertaken.

## Conclusion

The argument-based approach to validity is gaining attention in health-related measurement, with few practical examples of its application. This paper demonstrates how to apply the approach to develop a validity testing plan for the use of a widely used PROM in a new context. The phases and steps described support the uptake of this validity planning approach and promote scientific rigour in the field of health-related measurement. Importantly, the validity testing plan presented outlines the underpinning assumptions for the interpretation and use of HLQ scores in the NSW prison context. Moreover, it brings the theory into practice by offering a practical exemplar for identifying assumptions, evidence needs and methodological options to support validity testing. This example HLQ validity plan provides a practical guide for undertaking validity testing and generating relevant validity evidence to develop an evidence-based argument to verify score interpretation and use in a context.

## Supplementary Information

Below is the link to the electronic supplementary material.

Supplementary Material 1

## Data Availability

No datasets were generated or analysed during the current study.

## Abbreviations

PROM: Patient-reported outcome measure
HLQ: Health Literacy Questionnaire
NSW: New South Wales
Justice Health NSW: Justice Health and Forensic Mental Health Network
CSNSW: Corrective services New South Wales
The *Standards*: Standards for educational and psychological measurement
Ophelia: Optimising health literacy and access
IRT: Item response theory
CRC: Categorical response category
GPCM: Generalised partial credit model
BSEM: Bayesian structural equation modelling
CFA: Confirmatory factor analysis
