# Supplementary material for: Developing a validity testing plan for a patient-reported outcome measure in a new context using the argument-based approach to validity
Source: Qual Life Res. 2026 Jun 6;35(7):169. doi: 10.1007/s11136-026-04269-x (PMC13242489; doi:10.1007/s11136-026-04269-x)
Supplement: Supplementary file 1 — Supplementary Material 1 [file 11136_2026_4269_MOESM1_ESM.docx]

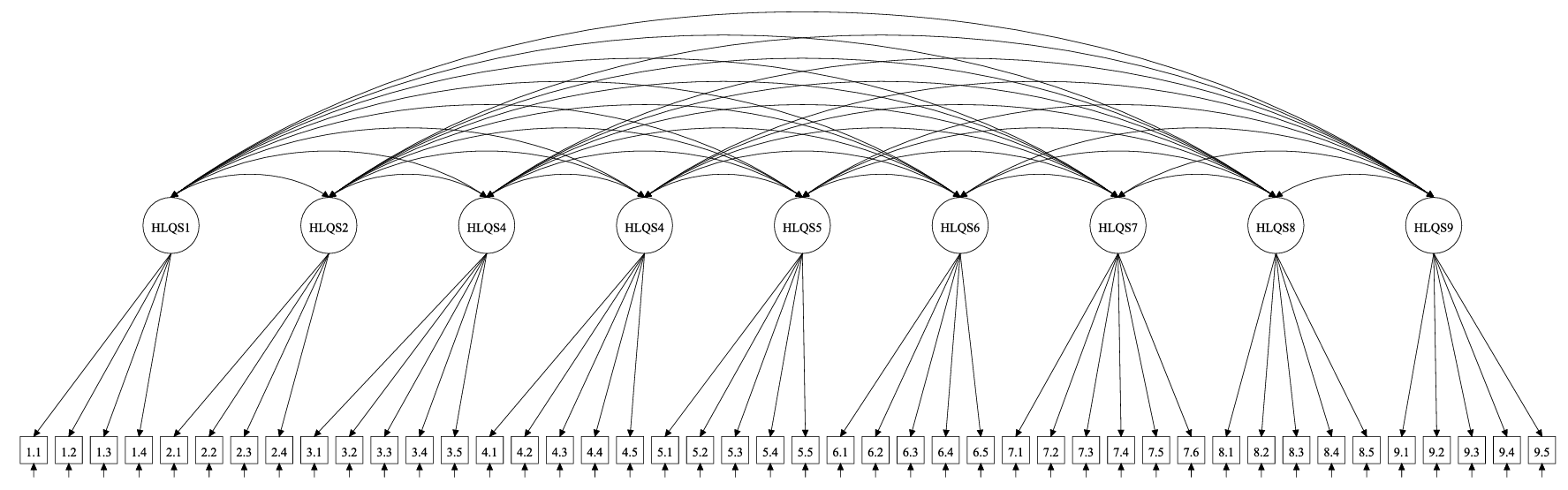


**Supplementary Figure 1: The nine-scale *a prior* HLQ conceptual structure.**

Note: HLQS1 = 1. Feeling understood and supported by healthcare providers; HLQS2 = 2. Having sufficient information to manage my health; HLQS3 = 3. Actively managing my health; HLQS4 = 4. Social support for health; HLQS5 = 5. Appraisal of health information; HLQS6 = 6. Ability to actively engage with healthcare providers; HLQS7 = 7. Navigating the healthcare system; HLQS8 = 8. Ability to find good health information; HLQS9 = 9. Understanding health information well enough to know what to do.
